# Magnetic Nanocomposite Scaffold-Induced Stimulation of Migration and Odontogenesis of Human Dental Pulp Cells through Integrin Signaling Pathways

**DOI:** 10.1371/journal.pone.0138614

**Published:** 2015-09-18

**Authors:** Hyung-Mun Yun, Eui-Suk Lee, Mi-joo Kim, Jung-Ju Kim, Jung-Hwan Lee, Hae-Hyoung Lee, Kyung-Ran Park, Jin-Kyu Yi, Hae-Won Kim, Eun-cheol Kim

**Affiliations:** 1 Department of Oral and Maxillofacial Pathology & Research Center for tooth and periodontal tissue regeneration (MRC), School of Dentistry, Kyung Hee University, Seoul, Korea; 2 Department of Oral and Maxillofacial Surgery, Guro Hospital, Korea University, Seoul, Republic of Korea; 3 Institute of Tissue Regeneration Engineering (ITREN), Dankook University, Cheonan, Republic of Korea; 4 Department of Nanobiomedical Science & BK21 PLUS NBM Global Research Center for Regenerative Medicine, Dankook University, Cheonan, Republic of Korea; 5 Department of Biomaterials Science, College of Dentistry, Dankook University, Cheonan, Republic of Korea; Texas A&M University Baylor College of Dentistry, UNITED STATES

## Abstract

Magnetism is an intriguing physical cue that can alter the behaviors of a broad range of cells. Nanocomposite scaffolds that exhibit magnetic properties are thus considered useful 3D matrix for culture of cells and their fate control in repair and regeneration processes. Here we produced magnetic nanocomposite scaffolds made of magnetite nanoparticles (MNPs) and polycaprolactone (PCL), and the effects of the scaffolds on the adhesion, growth, migration and odontogenic differentiation of human dental pulp cells (HDPCs) were investigated. Furthermore, the associated signaling pathways were examined in order to elucidate the molecular mechanisms in the cellular events. The magnetic scaffolds incorporated with MNPs at varying concentrations (up to 10%wt) supported cellular adhesion and multiplication over 2 weeks, showing good viability. The cellular constructs in the nanocomposite scaffolds played significant roles in the stimulation of adhesion, migration and odontogenesis of HDPCs. Cells were shown to adhere to substantially higher number when affected by the magnetic scaffolds. Cell migration tested by *in vitro* wound closure model was significantly enhanced by the magnetic scaffolds. Furthermore, odontogenic differentiation of HDPCs, as assessed by the alkaline phosphatase activity, mRNA expressions of odontogenic markers (DMP-1, DSPP,osteocalcin, and ostepontin), and alizarin red staining, was significantly stimulated by the magnetic scaffolds. Signal transduction was analyzed by RT-PCR, Western blotting, and confocal microscopy. The magnetic scaffolds upregulated the integrin subunits (α1, α2, β1 and β3) and activated downstream pathways, such as FAK, paxillin, p38, ERK MAPK, and NF-κB. The current study reports for the first time the significant impact of magnetic scaffolds in stimulating HDPC behaviors, including cell migration and odontogenesis, implying the potential usefulness of the magnetic scaffolds for dentin-pulp tissue engineering.

## Introduction

Regenerative endodontics aims to restore the function of pulp-dentin complex tissues mainly utilizing dental stem cells with the help of signaling molecules and scaffolding matrices. Scaffold is a three dimensional (3D) porous framework that serves as a potential biological carrier to facilitate repopulation of stem cells [1]. Among the scaffolding materials, natural polymers have excellent biocompatibility, yet they are mechanically fragile and often provoke immune responses [2]. On the other hand, synthetic polymers mainly those made of polyesters, including polylactic acid (PLA), polyglycolic acid (PGA), poly lactic-co-glycolic acid (PLGA), and poly-caprolactone (PCL), are degradable and tissue compatible, yet the cellular activity is still not satisfactory. [3–7].

While the polymeric materials can provide good scaffolding conditions for tissue regeneration, inorganic additives in nanoparticulate form including hydroxyapatite, tricalcium phosphate and bioactive glass, have been added to improve the mechanical and biological properties. Among the nanoparticulate additives, magnetic nanoparticles (MNPs) have recently gained great interest [8–10]. MNPs exhibit superparamagnetism and respond to magnetic fields; therefore, the addition of MNPs enables the scaffolds to exhibit magnetic properties. While the native form of MNPs has been shown poor water dispersibility and some cellular toxicity at high doses, the surface of MNPs has often been tailored with silica or surfactant to improve dispersibility and biocompatibility [11, 12].

Because of their intriguing properties, MNPs-added biomaterials have been intensively studied for the last a few years. Some of the recent works on MNPs-added biomaterials include MNP-hydroxyapatite ceramics, MNP-calcium phosphate cements and MNP-biopolymer scaffolds [13–16]. When MNPs were added to bioceramics the bone cell growth and differentiation have been improved [13]. The magnetic cements made of MNPs with alpha-tricalcium phosphate also showed stimulation in cellular adhesion and osteogenic differentiation [15]. Moreover, the addition of MNPs to biopolymer scaffolds increased adhesion and differentiation of osteoblast cells [15–17]. While those MNPs-added biomaterials have shown the potential in stimulation of cellular mitosis and ostogenic differentiation, the mechanism underlying the phenomena has rarely been disclosed. The magnetism-induced local mechano-activation of cells has been proposed as the possible molecular reason [15–16]. Moreover, the applications of the magnetic scaffolds to tissues other than bone have also been limited. It is however believed that the local magnetism induced in the magnetic scaffolds can affect the behaviors of a range of cells and tissues including dental specific cells.

Therefore, we focus our interest in the applications of the magnetic scaffolds for dental pulp-dentin regeneration. As a first step for this, here we investigate the effects of magnetic biopolymer scaffolds containing MNPs on the behaviors of human dental pulp cells (HDPCs) including adhesion, growth, migration and odontoblastic differentiation, and further explore the underlying signaling pathways involved in the cellular processes.

## Experimental Part

### Preparation of magnetic nanocomposite scaffolds

MNPs were synthesized using the method described previously [17,18]. Briefly, MNPs were fabricated by mixing 3.5318g Fe(acac)_3_ (iron(III) acetylacetonate), 3.9123g 1,2-hexadecanediol, 10ml oleic acid, 10ml oleylamine, and 40ml benzyl ether under a flow of nitrogen gas. The mixture was preheated to reflux at 200°C for 30 minutes during stirring, and subsequently for an additional 2 hours, heated to 300°C under a nitrogen atmosphere. The black-brown mixture was cooled to room temperature and 50ml ethanol added. The products were collected following centrifugation at 10000 rpm for 5 minutes, washed four times with ethanol, and dried at 50°C overnight.

For the preparation of PCL+MNPs scaffolds, 10% w/v of PCL (~80 kDa, Sigma-Aldrich, USA) was dissolved in chloroform, and then the prepared MNPs were added. The concentrations of MNPs in the PCL solutions were set at 0, 5, and 10%wt, which we have designated as PCL, PCL+MNPs 5%, and PCL+MNPs 10%, respectively. After sieving (200–500 mm in diameter), the NaCl particles were poured into a cylindrical plastic mould and packed tightly. The solutions were ultrasonicated and added dropwise to the NaCl-filled mould, followed by freezing at -70°C and freeze-drying for 3 days. The resulting samples were washed nine times with distilled water in order to leach out the salt completely, and then dried again. All of the scaffolds were sterilized with ethylene oxide gas.

### Characterization of the scaffolds

The nanoscale morphology of the MNPs was observed by transmission electron microscopy (TEM, 7100 microscope, JEOL, USA). The surface morphologies of the scaffolds were observed using scanning electron microscopy (SEM; S-3000H Hitachi, Japan), and atomic composition was analyzed using energy dispersive spectroscopy (EDS). The crystal structures were determined by X-ray diffraction (Rigaku, Danvers, MA, USA). Specimens were scanned in the range of two theta diffraction angles from 10 to 60° at a rate of 2° min^−1^, with a step width of 0.02° using Cu Kα1 radiation at 40kV and 40mA current strength. Fourier transformed infrared (FT-IR; Perkin-Elmer, USA) spectroscopy was used to observe the chemical binding state of the scaffolds. The magnetic properties of the scaffolds were examined using a vibrating sample magnetometer (VSM; Quantum Design MPMS-XL7, USA) within the applied magnetic field range of ± 20 kOe at room temperature [15], in terms of the saturation magnetization and hysteresis loop.

### Cell culture

Immortalized HDPCs with the telomerase catalytic subunit of the human telomerase reverse transcriptase (hTERT) were kindly provided by Professor Takashi Takata (Hiroshima University, Japan), and a first described article for the immortalized HDPCs that were performed in compliance with regulations administered by the experimentation committee of the Graduate School of Biomedical Sciences, Hiroshima University was cited. [19]. Cells were cultured in α-MEM supplemented with 10% FBS, 100U/ml penicillin, and 100μg/ml streptomycin in a humidified atmosphere of 5% CO_2_ at 37°C. HDPCs were seeded onto PCL+MNPs scaffolds already placed in 24-well tissue culture plates. To induce differentiation, HDPCs were cultured in osteogenic supplement (OS, 10mM β-glycerophosphate and 50mg/ml ascorbic acid) as described previously [20–23]. Direct seeding of cells (3×10^5^ cells/ 6 well plate) was performed on the PCL and PCL+MNPs scaffolds. The media was changed every two days.

### Cell viability on scaffolds

Cell viability on scaffolds was confirmed using a 3-(4,5-dimethylthiazol-2-yl)-5-(3-carboxymethoxyphenyl)-2-(4-sulfophenyl)-2H-tetrazolium (MTS) assay kit (Cell Titer 96 Aqueous One Solution; Promega, Madison, WI) following the protocol suggested by the manufacturer. HDPCs were seeded on the samples at a density of 3x10^5^ cells/well of a 6-well culture plate, and cell growth was measured using a microplate absorbance reader at a wavelength of 490nm. Cells were observed after fixation in ice-cold 4% paraformaldehyde in PBS for 20 minutes and subsequently permeabilization with 0.1% Triton X-100 in PBS for 10 minutes at 4°C. The F-actin filaments and p65 (Santa Cruz Biotechnology, Santa Cruz, CA) were stained with rhodamine phalloidin (Invitrogen, CA), and nuclei were stained with 10μg/ml propidium iodide. The samples were visualized using confocal microscopy (Cell Voyager, Yokogawa,Japan).

### Cell adhesion assay

50μl ECM gel solution (Cell Biolabs, San Diego, CA) was poured onto a 96-well culture plate and allowed to solidify (37°C, 1 hour). HDPCs (1 × 10^4^ cells/well) that were cultured in the presence or absence of scaffolds for three days were seeded onto 96-well plates and allowed to attach at 37°C for the indicated times. Adherent cells were fixed with 10% paraformaldehyde for 30 minutes at room temperature, and stained with 0.5% crystal violet for 10 minutes. The cell-bound stain was dissolved by incubating the wells with 1% SDS overnight in the dark. The optical density was obtained at 595 nm with a microplate reader.

### Cell migration assay

Cell migration was accessed using an *in vitro* scratch assay. HDPCs (1 × 10^4^ cells/well) were seeded onto 6-well plates and allowed to grow to 90% confluence at 37°C under a 5% CO_2_ atmosphere. Cell monolayers were wounded with a 200 μl pipette tip and washed three times with PBS to remove cell debris. HDPCs were incubated in the presence or absence of scaffolds for 12 hours, and the images were compared in order to quantify the migration rate of the cells.

### Odontogenic differentiation assays

Cells were washed with PBS, and sonicated using a cell disruptor. As the indication of odontogenic differentiation, the alkaline phosphatase (ALP) activity was first determined using *p*-nitrophenyl phosphate (3mM final concentration) as the substrate in 0.7 M 2-amino-methyl-1-propanol, pH10.3, and 6.7 mM MgCl_2_. Absorbance was measured at 410 nm using an enzyme-linked immunosorbent assay reader (Beckman Coulter, Fullerton, CA).

Next, the mRNA expressions of odontogenic genes including dentin matrix protein-1 (DMP-1), dentin sialo phosphoprotein (DSPP), ALP, osteocalcin, and ostepontin were examined. For this, total RNA was extracted using TRIzol reagent (Life Technologies, Gaithersburg, MD) according to the manufacturer instructions and 1 μg reagent (Life Technologiribed for first-strand cDNA synthesis (Gibco BRL, Rockville, MD). The cDNA was amplified in a total volume of 20 μl was with 2.5 mM magnesium dichloride, 1.25 units Ex Taq polymerase (Bioneer, Daejeon, Korea) and 1μM specific primers. Amplification was carried out for 30 cycles in a thermal cycler. The primer sequences for each gene are listed in Table 1. PCR products were resolved on a 1.5% agarose gels, stained with ethidium bromide, and analyzed under the ChemiDoc XRS+ imaging system with Image Lab image acquisition and analysis (Bio-Rad, Hercules, CA).

**Table 1:** Reverse transcriptase-polymerase chain reaction (RT-PCR) primers and conditions.

Genes	Primer Sequence (5'-3')	Annealing Temp (℃)	Cycle Number	Product Size (bp)
ALP	F: 5'-CGAGTGCAGCTCATACTCCATGC-3'	60	35	507
	R: 5'-CCGCGTGTCGTGTTGCACTG-3'			
OPN	F: 5'- CCCACAGACCCTTCCAAGTA-3'	60	35	196
	R: 5'- GGGGACAACTGGAGTGAAAA-3'			
OCN	F: 5'-GTGCAGCCTTTGTGTCCAAGCAGGA-3'	60	30	244
	R: 5'-CCGTAGAAGCGCCGATAGGCC-3G			
DSPP	F: 5'- AGAAGGACCTGGCCAAAAAT-3'	60	35	280
	R: 5'- TCTCCTCGGCTACTGCTGTT -3A			
DMP-1	F: 5'- CAGGAGCACAGGAAAAGGAG -3'	56	36	213
	R: 5'-CTGGTGGTATCTTCCCCCAGGAG -33			
Integrin nt	F: 5'- TGCTGCTGGCTCCTCACTGTTGT -3'	65	35	703
	R: 5'- TAGTCTGGCGGCCACCTCTCTG -33			
Integrin nt	F: 5'- TTTCCCTGCTCTCACCGGGC -3'	62	32	132
	R: 5'- ACCGGGGGACCGTAGTTGCG -3'			
Integrin nt	F: 5'- GACGCCGCGCGGAAAAGATG -3'	60	30	244
	R: 5'- ACCACCCACAATTTGGCCCTGC -33			
Integrin nt	F: 5'-CCGGCCAGATGATTCGAAGA-3'	55	35	159
	R: 5'-GGGTCACCTGGTCAGTTAGC-3'			
β -actin	F: 5'- CATGGATGATGATATCGCCGCG-3'	60	35	371
	R: 5'- ACATGATCTGGGTCATCTTCTCG-3'			

For the mineralization assay, cells fixed with 95% methanol were stained with 1% Alizarin Red for 5 minutes, washed with PBS, and observed under a microscope.

### Signaling pathway studies

For the signaling studies, the mRNA levels of the adhesion ligand, integrin subsets, including subsets, Integrin α1: 5'-TGCTGCTGGCTCCTCACTGTTGT-3' (sense) and 5'-TAGTCTGGCGGCCACCTCTCTG-3' (anti-sense), Integrin α2: 5'-TTTCCCTGCTCTCACCGGGC-3' (sense) and 5'-ACCGGGGGACCGTAGTTGCG-3' (anti-sense), Integrin β1: 5'-GACGCCGCGCGGAAAAGATG-3' (sense) and 5'-ACCACCCACAATTTGGCCCTGC-3 (anti-sense).

The Western blot analysis was used to investigate the protein expressions of intracellular adhesion molecules, including paxillin, focal adhesion kinase (FAK), and RhoA, as well as the MAPK, which is the integrin downstream signaling protein and consists of extracellular signal-regulated kinase (ERK), c-Jun amino-terminal kinase (JNK) and p38. Furthermore, the protein expressions of nuclear factor of kappa light polypeptide gene enhancer in B-cells inhibitor, alpha (IκBα), and the nuclear factor kappa-light-chain-enhancer of activated B cells (NFκBp65) were analyzed. β-actin was used as the reference protein.

The total protein was extracted from HDPCs using ice-cold 1% Triton X-100 lysis buffer. Equal amounts of protein were separated on 12% gels using sodium dodecyl sulfate polyacrylamide gel electrophoresis (SDS-PAGE), followed by transfer to polyvinylidene fluoride (PVDF) membranes. Membranes were probed with specific antibodies (1:1000), followed by incubation with a secondary horseradish peroxidase-conjugated antibody (1:100000). Proteins were detected by an enhanced chemiluminescence system (Amersham, Piscataway, NJ) according to the manufacturer’s instructions.

### Statistical analysis

Data are presented as the mean ± standard deviation. The statistical analysis of the data was performed by one-way analysis of variance followed by a multi-comparison Tukey’s test using the SPSS program (SPSS 22.0, IBM, Armonk, NY, USA). Statistical significance was determined at p < 0.05.

## Results

### Characteristics of magnetic scaffolds

**Fig 1:**
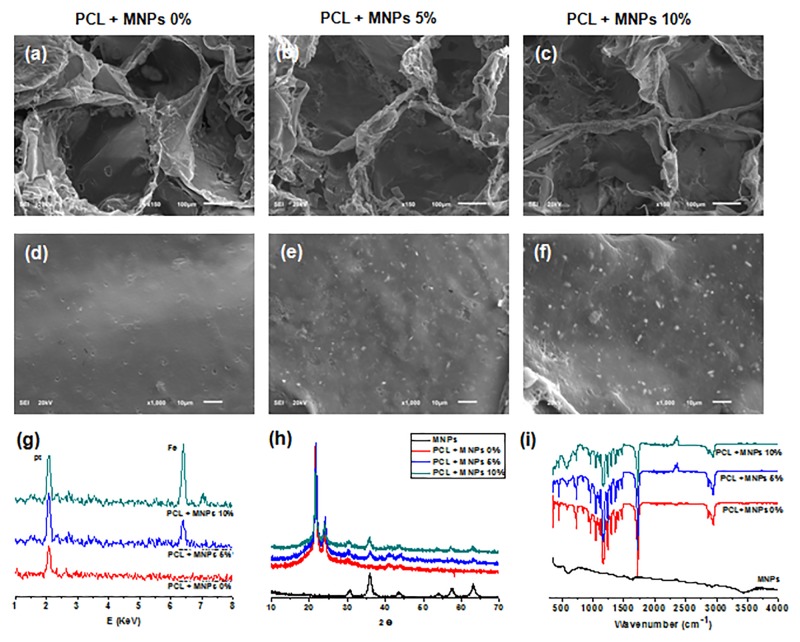
Characteristics of magnetic scaffolds: (a-f) SEM images at different magnifications, (g) EDS atomic signal, (h) XRD phase analysis and (i) FT-IR chemical groups. Low (a-c, 150 X) and high (d-f, 1000 X) SEM morphologies of PCL, PCL–MNPs 5%, and PCL–MNPs 10% scaffolds showing a highly porous structure, and their EDS results showing existence of MNPs (Fe) on the surface, which was also confirmed by XRD and FT-IR spectrum.

The MNPs prepared by a reflux method revealed homogeneous nanoparticles from TEM image (S1a Fig). All the scaffolds either incorporating the MNPs or not revealed a well-developed pore structure without a significant difference in the pore morphology or size (250–500 μm), as shown in Fig 1a–1c. With increasing MNPs content in the scaffold, the surface became rougher due to presence of MNPs on the surface (Fig 1d–1f). The Fe peak was detected only in the PCL+MNPs from EDX analysis (Fig 1g). From XRD phase analysis, the PCL+MNPs showed different diffraction peaks at 2θ of 31, 36, 43, 54, 57, and 63, typical of bulk magnetite Fe_3_O_4_, compared with PCL alone (Fig 1h). This peak intensity became higher in the PCL+MNPs as the MNPs content increased, being in good agreement with the EDX data [22]. The chemical bond structure of the scaffolds, as revealed by the FT-IR spectrum (Fig 1i), shows typical bands related to PCL at 1720 cm^-1^ (C = O stretching), 1293 cm^-1^ (C-O stretching) and 3153–3640 cm^-1^, which represent the alcohol groups of PCL. The peak at 578 cm^-1^ assigned to the Fe–O bond vibration of MNPs was only detected in PCL+MNPs [23,24]. This band became sharper in the PCL+MNPs as the MNPs content increased. The magnetic properties of the scaffolds, as examined by the vibrating sample magnetometer, revealed hysteresis loop which is typical of magnetic materials (S1b Fig). The saturation magnetization was recorded to be 1.63 and 3.02 emu/g, respectively, for the scaffolds containing 5% and 10%MNPs.

### Cell viability and population in magnetic scaffolds

**Fig 2:**
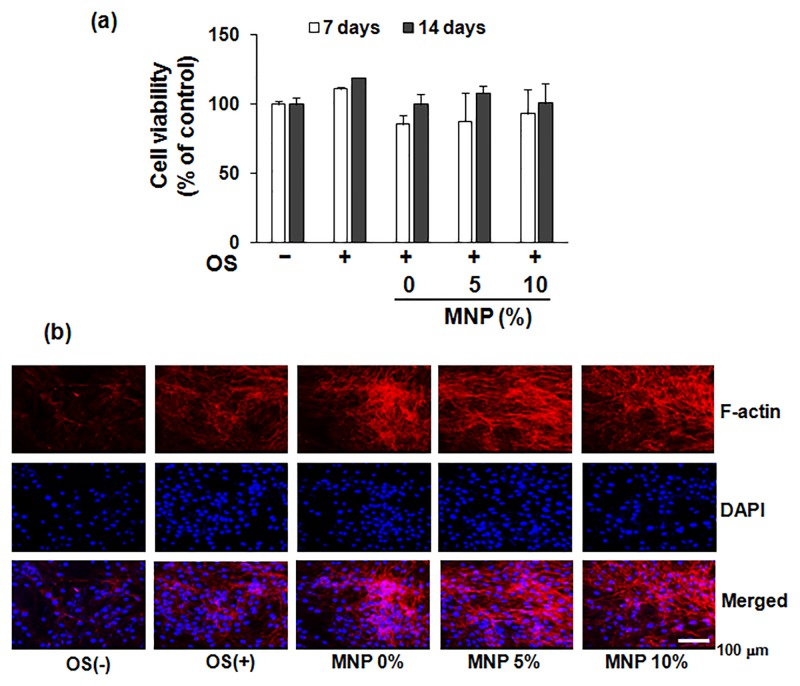
HDPCs population and viability over the magnetic scaffolds. (a) Cell viability results measured by MTS for up to 14 days, and (b) cytoskeletal processing images at day 3. Representative confocal microscopy images of cells stained for F-actin with rhodamine phalloidin (red staining) and nuclei were stained with DAPI (blue staining). OS: odontogenic supplements.

The prepared magnetic scaffolds were shown to populate HDPCs actively, as shown in Fig 2. MTS cell viability assay revealed no substantial time-dependent down-regulation in cell viability upon the magnetic scaffolds (Fig 2a). The cell viability was comparable between the scaffold groups. The F-actin cytoskeleton processes, as observed by fluorescence microscopy at day 3, were active on the scaffolds. The cellular cytoskeletal development was also comparable between the scaffold groups (Fig 2b).

### Effects on the cell adhesion and migration

**Fig 3:**
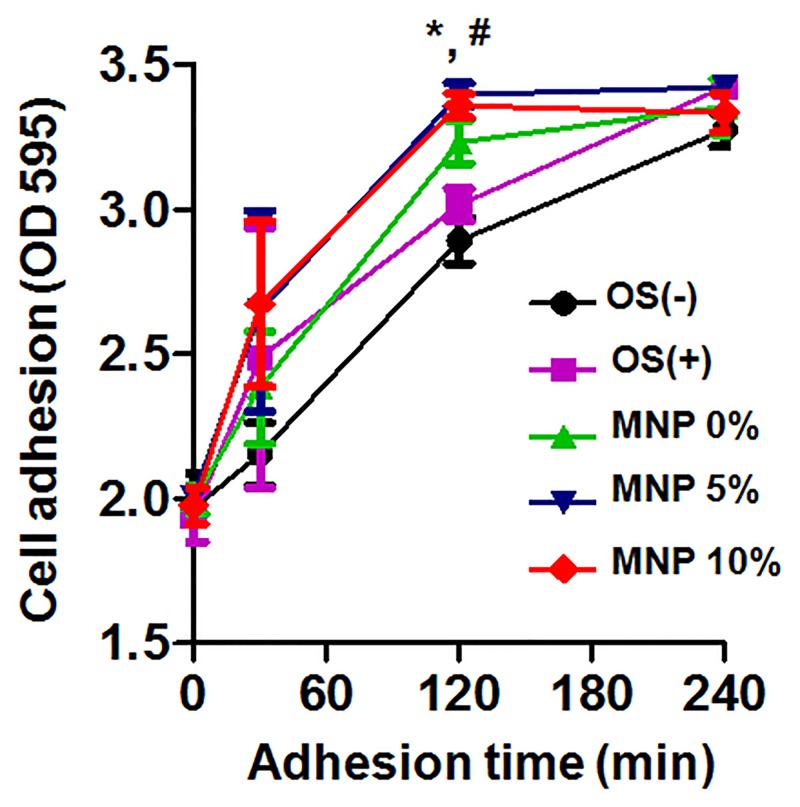
Effects of magnetic scaffolds on the adhesion of HDPCs for up to 240 min. HDPCs was fixed and stained at 30, 120, and 240 min. After dissolving the stained cells, the optical density was obtained at 595 nm. These data findings are representative of three independent experiments. *: statistically significant difference compared with OS (p<0.05, n = 4). #: statistically significant difference compared with 0% MNP (p<0.05, n = 4).

The cellular constructs implemented on the scaffolds for 3 days affected the HDPCs’ adhesion and migration ability. The cell adhesion study made on Matrigel coating for 120 min showed that the treatment with magnetic scaffolds significantly stimulated the cell adherence number (Fig 3). Interestingly, the cells cultured on non-magnetic pure PCL scaffolds even improved the HDPCs adhesion, when compared to the 2D cultures (both non-odontogenic and odontogenic medium).

**Fig 4:**
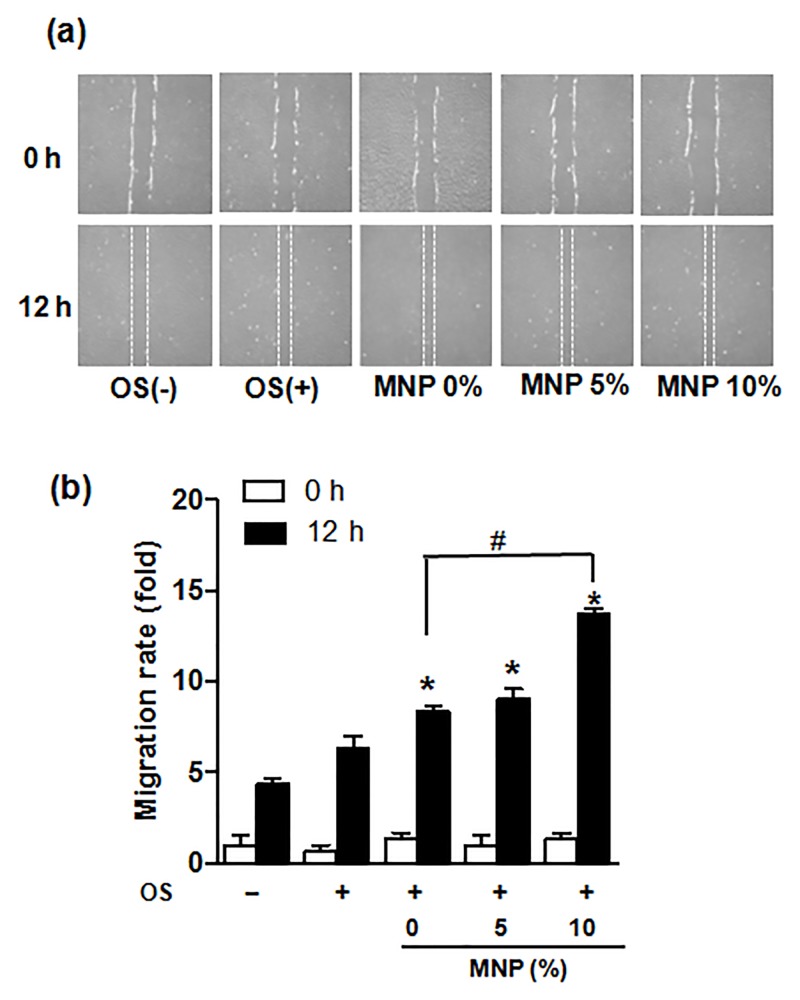
Effects of magnetic scaffolds on the migration of HDPCs; (a) cell images before and after migration for 12 hours, and (b) migration rate measured. *: statistically significant difference compared with 0%MNP (p<0.05, n = 3).

Cell migration assay, studied using in vitro wound closure model, showed the HDPCs migration for 12 h was enhanced significantly by the scaffold groups, and even more enhanced when treated with magnetic scaffolds which also in a MNP dose-dependent manner (Fig 4).

### Effects on the odontogenic differentiation

**Fig 5:**
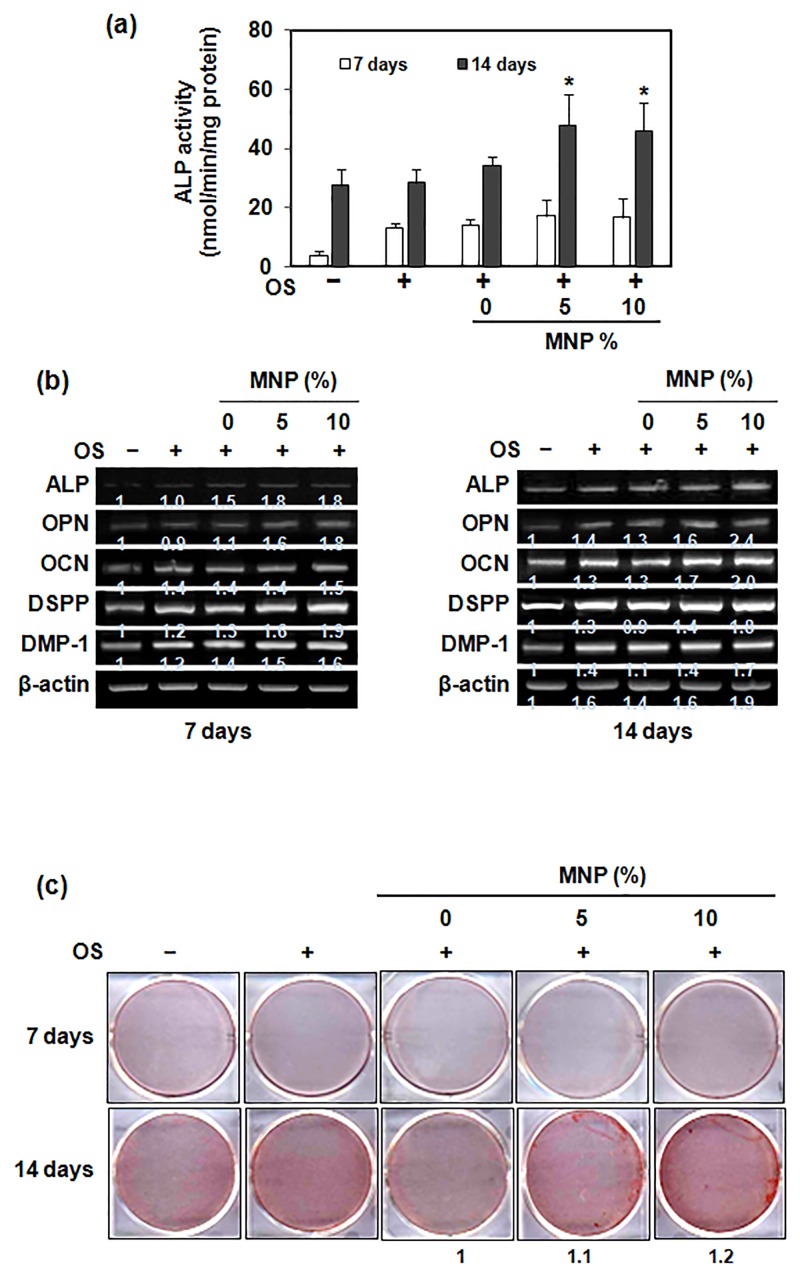
Effects of magnetic scaffolds on the odontogenic differentiation of HDPCs. Differentiation was determined by (a) alkaline phosphatase (ALP) activity, (b) mRNA expression of genes (ALP, OPN, OCN, DSPP, and DMP-1) by RT-PCR, and (c) mineralization nodule formation by Alizarin red staining. *: statistically significant difference compared with 0%MNP (p<0.05, n = 3).

To evaluate the effects of the magnetic scaffolds on the odontoblastic differentiation of HDPCs, the ALP activity, mRNA expression of odontogenic markers, and mineralized nodule formation were assessed (Fig 5). The incorporation of MNPs significantly increased ALP activity, particularly at day 14 (Fig 5a). The odontogenic expressions including ALP, OPN, OCN, DMP-1, and DSPP were also substantially enhanced by the magnetic scaffolds (Fig 5b). The mineralized nodule formation, as analyzed by ARS method, was much clearer in the magnetic scaffold groups (Fig 5c). However, PCL+MNPs scaffolds themselves without OS did not affect the odontogenesis of HDPCs (S2 Fig).

### Signal transduction pathways

**Fig 6:**
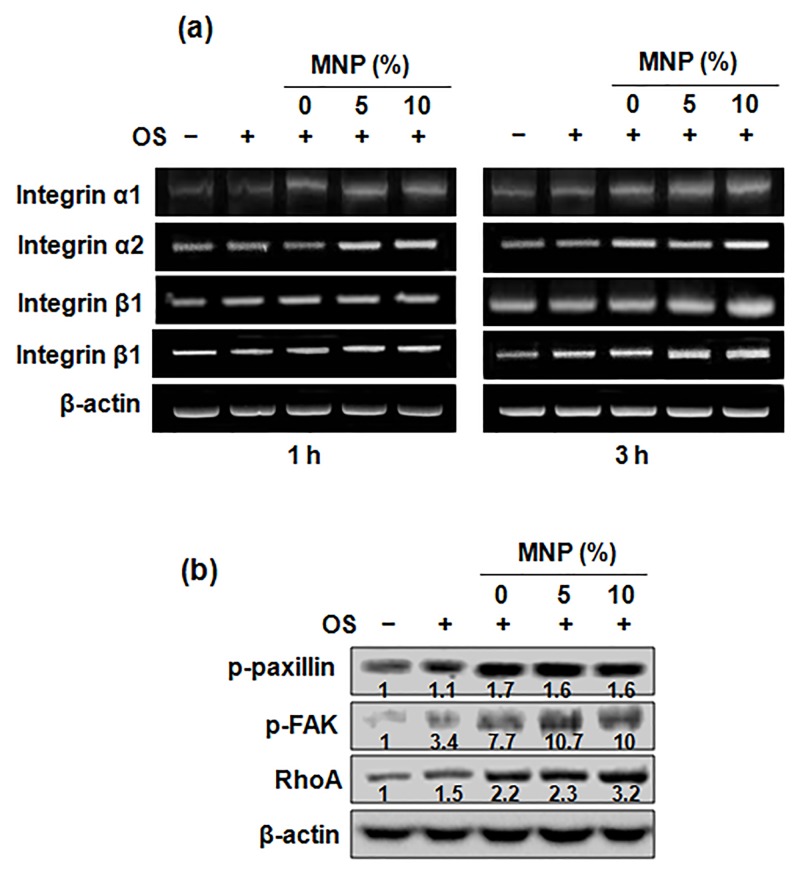
(a) Gene expressions of integrin subsets as analyzed by RT-PCR at 60 min and 180 min and (b) the protein expressions involved in integrin downstream pathways as assessed by Western blotting at 60 min. These data are representative of three independent experiments.

To examine whether the magnetic scaffolds activate integrin-mediated signaling pathways, the mRNA expression of integrin and its downstream pathways were examined at the very early time points (1 and 3 hours). Expression of integrin α1, α2, β1 and β3 mRNA increased for the magnetic scaffolds compared with those for PCL alone (Fig 6a). In addition, the phosphorylation of paxillin and focal adhesion kinase (FAK), and the expression of RohA gene were enhanced by the magnetic scaffolds (Fig 6b).

**Fig 7:**
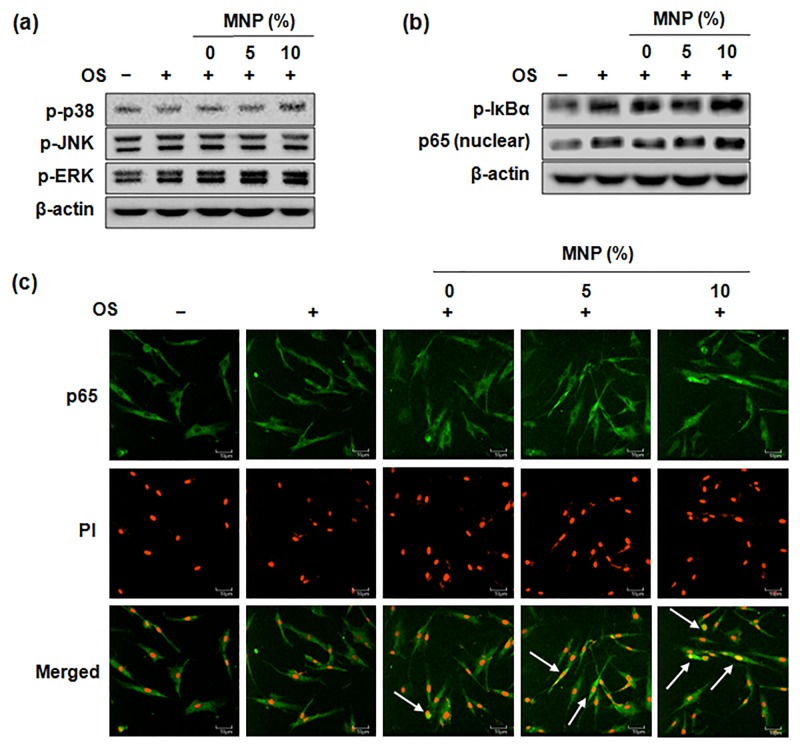
Activation of intracellular integrin downstream pathways: (a) MAPK and (b, c) NF-κB. Cells were cultured for (a) 45 minutes and (b, c) 60 minutes. Signaling pathways were assessed via (a, b) Western blotting and (c) immunofluorescence staining. A change in the color of the nucleus from red to yellow (due to co-localization of green FITC fluorescence and red propidium iodide fluorescence, arrows) was indicative of NF-κB translocation in the cells. These data are representative of three independent experiments.

To investigate further whether the signal caused by the magnetic scaffolds was through the MAPK and NF-κB pathway, the activation of MAPK and NF-κB of HDPCs in the scaffold was examined by Western blotting analysis and immunofluorescence. ERK and p38 phosphorylation were higher in HDPCs by the magnetic scaffolds, however the phosphorylation of JNK appeared not to be significantly affected (Fig 7a). All scaffolds resulted in the phosphorylation of IκBα and nuclear translocation of p65, and the expression levels were higher by the magnetic scaffolds as compared to those by PCL (Fig 7b). Similarly, the immunofluorescence detection of NF-κB p65 in the nucleus increased in the magnetic scaffolds compared with PCL scaffold (Fig 7c).

## Discussion

This study clearly demonstrated the significant effects of the magnetic scaffolds on the behaviors of HDPCs, and to the best of our knowledge this is the first report on utilizing the magnetic scaffolds for dental pulp-dentin regeneration purposes. This type of porous foam scaffolds can be potentially useful for the regenerative therapy of dentin-pulp complex tissues, where the pulp stem cells can be recruited through the scaffolding materials to anchor to, populate on, and further to differentiate into an odontogenic lineage [25–27].

The pore structure of the magnetic scaffolds implemented herein is considered to be favorable for use as tissue engineering 3D matrices. In general, macropores larger than 250 μm, while those smaller than 100 μm can restrict cellular infiltration and migration through the pores [28]. In fact, pores larger than 300 μm have been shown to improve the osteogenesis and odontogenesis of cells [28–30]. The pores ranging from 250 to 500 μm determined in this scaffold are thus considered to be suitable for cellular population and odontogenic differentiation [17,28]. Here, the scaffolds were active in populating HDPCs throughout the pore space, preserving the cell viability over 14 days. In terms of the mechanical properties of the magnetic scaffolds, the addition of MNPs has previously been shown to enhance the compressive strength and elastic modulus [15]. The improved mechanical properties, particularly elastic modulus values, are considered to be a favorable aspect of 3D matrices when one aims to drive stem cells (HDPSCs) to differentiate into an odontogenic lineage.

When HDPCs were cultivated on the magnetic scaffolds, their effects on cellular adhesion and migration were substantial. The cell adhesion assay revealed significant anchorage of cells at the very initial culture periods (up to 3 h) when affected by the scaffolds. Even more, the magnetic scaffolds stimulated the cellular anchorage with respect to the non-magnetic pure PCL counterpart. More intriguing result was shown on the cell migration ability, as assessed from the in vitro wound closure model. Cells narrow the gap almost completely at the time of 12 h culture when affected by the magnetic scaffolds. From the cell adhesion and migration studies, the scaffold itself appeared to improve the cellular behaviors, and adding to this, the effects of magnetism. It is considered that 3D culture of cells had some positive impact on cellular properties in a way that also secreting molecules like growth factors and cytokines that are beneficial for the cell adhesion and migration. Adding to this, the magnetism-induced cellular alteration should even more stimulate those signaling molecular secretions. At large the cells appeared to be more viable and active when affected by the magnetic 3D scaffolds [31].

Not only the cellular adhesion and migration, the odontogenic differentiation of HDPCs was significantly influenced. While ALP is relatively an early differentiation marker that increases during the proliferation and matrix syntheses stage [32], the ARS is a method to detect matrix calcium deposition, the latest step in odontoblast maturation [33]. Further, the genes including DSPP, DMP-1, OCN and OPN are known to be identifying markers of odontoblasts [20–23]. Therefore, the series of odontogenic differentiation assays, involving ALP activity, expressions of a set of odontogenic genes, and the ARS mineralization, clearly demonstrate the significant role of the 3D magnetic scaffolds played in accelerating odontogenesis of HDPCs

Since here we could observe the simultaneous stimulation in cell adhesion and odontogenic differentiation when influenced by the magnetic scaffolds, we hypothesized the molecular mechanism in the odontogenic stimulation could largely originate from the adhesion events. The adhesion events have been shown to determine subsequent cellular fate [34,35]. Therefore, we further analyzed the adhesion molecular processes. The key cell receptor integrin sets were first analyzed. HDPSCs are known to express the integrins specific to adhesion ligands in ECM. For example, the integrin α1β1 and α2β1 are specific to collagen, while αVβ3 being to bone sialoprotein, fibronectin, fibrinogen, and laminin [36,37]. We could observe the series of integrin subsets including α1, α2, β1, and β3 being highly up-regulated by the magnetic scaffolds. Mechanistically, integrin-mediated attachment activates FAK by auto-phosphorylation at Y-residues providing a binding site for Src. In turn, Src binds to a number of signaling molecules such as paxillin, which links the integrin-FAK signaling complex to the actin cytoskeleton [38]. Here, the subsequent expressions of phosphorylated FAK and paxillin were also up-regulated by the magnetic scaffolds, demonstrating the HDPCs cultured by the magnetic scaffolds were activated to express integrins α1, α2, β1 and β3 and the intracellular downstream signal pathways.

The MAPK and NF-κB signaling has been implicated as a downstream of integrin and an important cellular signal transduction pathway for odontogenesis in HDPSCs [39, 40]. Our findings here demonstrate that the phosphorylation of p38 and ERK and the activation of NF-κB were enhanced notable by the magnetic scaffolds, which further indicates that magnetic scaffolds may act through the integrin, FAK, MAPK pathway to induce NF-κB activation in HDPCs.

In summary, this study reports for the first time that the magnetic scaffolds made of PCL-MNP nanocomposite have significant implication in odontogenic differentiation of HDPCs. It is also demonstrated that integrin signaling downstream pathways with FAK/MAPK and NF-κB activation are involved in the cellular events by the PCL-MNP scaffolds. The investigated magnetic nanocomposite scaffolds are thus considered to provide excellent matrix conditions for HDPCs in their migration, adhesion, and odontogenic differentiation, the behaviors ultimately useful for scaffold-based dentin-pulp tissue engineering, which though warrants further in vivo confirmation in relevant animal models.

## Supporting Information

S1 Fig (a) TEM image of MNPs, revealing the generation of well-dispersed uniform-sized nanoparticles. (b) Magnetic properties of the PCL-MNPs scaffolds incorporating 5 and 10% MNPs, measured by VSM. Magnetization under applied magnetic field showed hysteresis loop with saturation magnetization of 1.63 and 3.02 emu/g, respectively for the scaffolds containing 5% and 10%MNPs. Data from previous work with slight modifications [15].
(TIF)

S2 Fig Effects of magnetic scaffolds themselves on the odontogenic differentiation of HDPCs. In the absence of OS, odontogenic differentiation by magnetic scaffolds themselves was determined mRNA expression of genes (OPN, OCN, and DMP-1) using RT-PCR (a), and alkaline phosphatase (ALP) activity, (c) mineralization nodule formation by Alizarin red staining at 7 and 14 days.
(PPT)
