# Supplementary figures and images for: Magnetic Nanocomposite Scaffold-Induced Stimulation of Migration and Odontogenesis of Human Dental Pulp Cells through Integrin Signaling Pathways
Source: PLoS One. 2015 Sep 18;10(9):e0138614. doi: 10.1371/journal.pone.0138614 (PMC4575126; doi:10.1371/journal.pone.0138614)

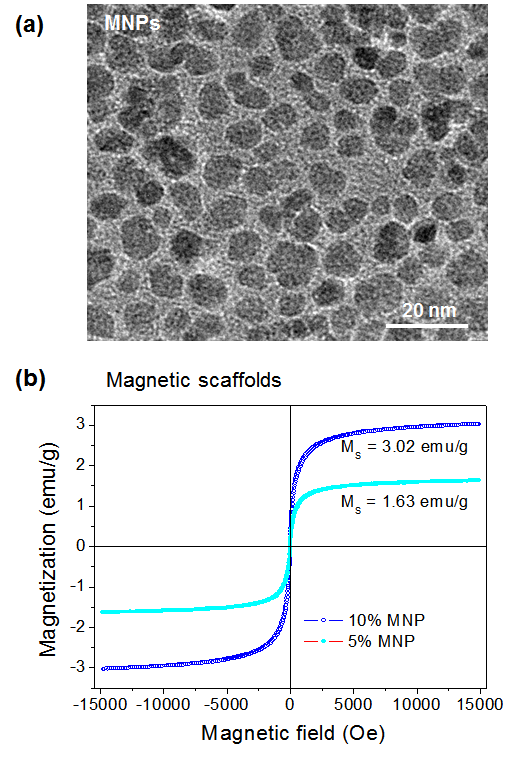

Supplement: S1 Fig — (a) TEM image of MNPs, revealing the generation of well-dispersed uniform-sized nanoparticles. (b) Magnetic properties of the PCL-MNPs scaffolds incorporating 5 and 10% MNPs, measured by VSM. Magnetization under applied magnetic field showed hysteresis loop with saturation magnetization of 1.63 and 3.02 emu/g, respectively for the scaffolds containing 5% and 10%MNPs. Data from previous work with slight modifications [15]. (TIF) [file pone.0138614.s001.tif]
