# Supplementary material for: Magnetic Nanocomposite Scaffold-Induced Stimulation of Migration and Odontogenesis of Human Dental Pulp Cells through Integrin Signaling Pathways
Source: PLoS One. 2015 Sep 18;10(9):e0138614. doi: 10.1371/journal.pone.0138614 (PMC4575126; doi:10.1371/journal.pone.0138614)
Supplement: S2 Fig — In the absence of OS, odontogenic differentiation by magnetic scaffolds themselves was determined mRNA expression of genes (OPN, OCN, and DMP-1) using RT-PCR (a), and alkaline phosphatase (ALP) activity, (c) mineralization nodule formation by Alizarin red staining at 7 and 14 days. (PPT) [file pone.0138614.s002.ppt]

## Slide 1
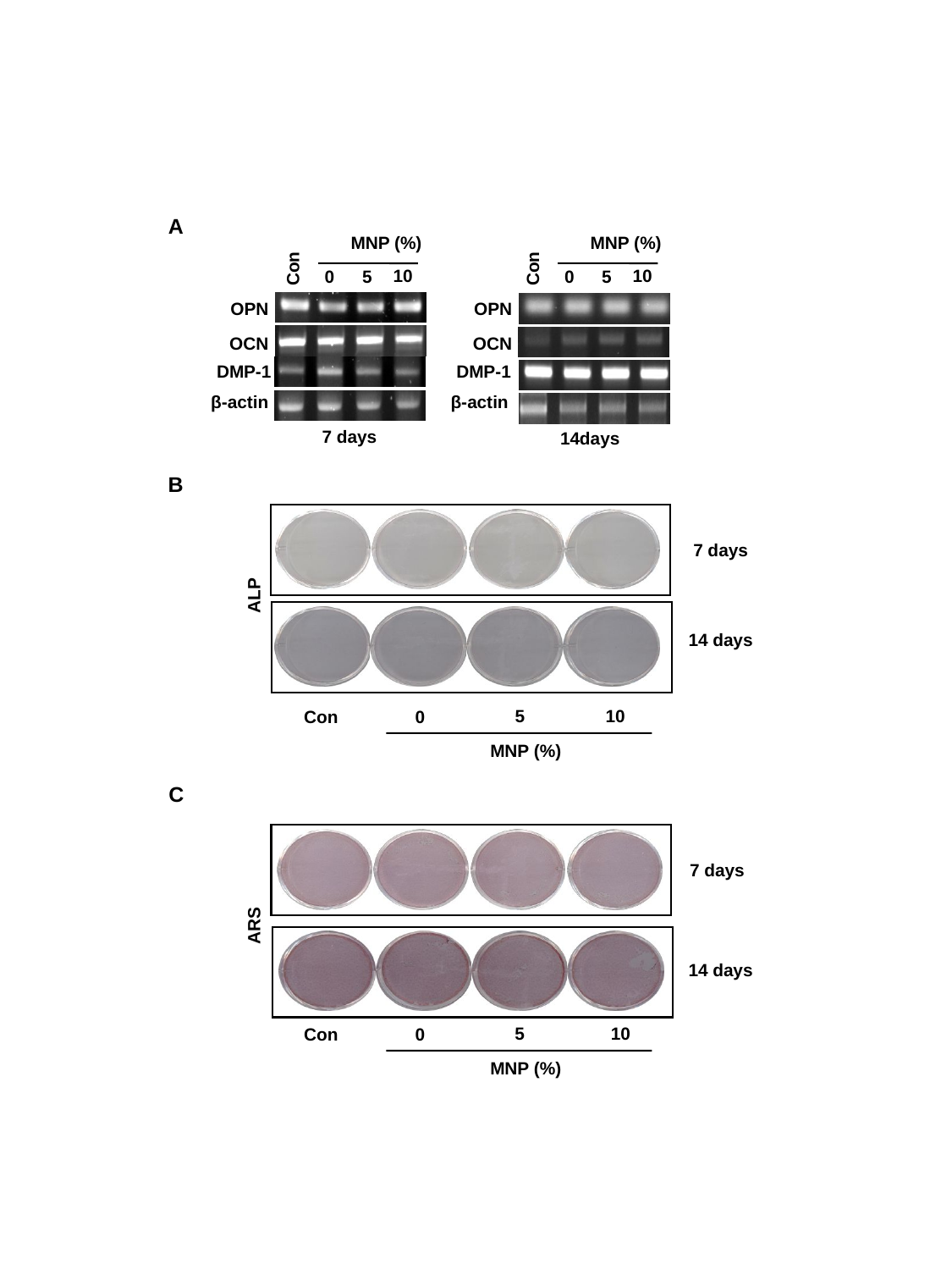

A
MNP (%)
MNP (%)
Con
Con
10
10
5
5
0
0
OPN
OPN
OCN
OCN
DMP-1
DMP-1
β-actin
β-actin
7 days
14days
B
7 days
ALP
14 days
10
5
Con
0
MNP (%)
C
7 days
ARS
14 days
10
5
Con
0
MNP (%)
